# Mycophenolic acid counteracts B cell proliferation and plasmablast formation in patients with systemic lupus erythematosus

**DOI:** 10.1186/ar3835

**Published:** 2012-05-09

**Authors:** Sebastian Eickenberg, Eva Mickholz, Elisabeth Jung, Jerzy-Roch Nofer, Herrmann Pavenstädt, Annett M Jacobi

**Affiliations:** 1 Rheumatology and Clinical Immunology Unit of the Department of Internal Medicine D, University Hospital Münster, Albert Schweitzer Str. 33, 48149 Münster, Germany; 2 Department of Internal Medicine D, University Hospital Münster, Albert Schweitzer Str. 33, 48149 Münster, Germany; 3 Center of Laboratory Medicine, University Hospital Münster, Albert Schweitzer Str. 33, 48149 Münster, Germany

## Abstract

**Introduction:**

Clinical trials revealed a high efficacy of mycophenolate mofetil (MMF) in inducing and maintaining remission in patients with class III-V-lupus nephritis. Also extrarenal manifestations respond to MMF treatment. However, few attempts have been undertaken to delineate its mechanism of action in systemic lupus erythematosus (SLE) a disease characterized by enhanced B cell activation.

**Methods:**

Clinical and paraclinical parameters of 107 patients with SLE were recorded consecutively and analyzed retrospectively. Patients were divided into treatment groups (MMF: n = 39, azathioprine (AZA) n = 30 and controls without immunosuppressive therapy n = 38). To further delineate the effect of mycophenolic acid (MPA) on naive and memory B cells in vitro assays were performed.

**Results:**

Although patients taking AZA flared more frequently than patients on MMF or controls, the analysis of clinical parameters did not reveal significant differences. However, profound differences in paraclinical parameters were found. B cell frequencies and numbers were significantly higher in patients taking MMF compared to those on AZA but lower numbers and frequencies of plasmablasts were detected compared to AZA-treated patients or controls. Notably, MMF treatment was associated with a significantly higher frequency and number of transitional B cells as well as naive B cells compared to AZA treatment. Differences in T cell subsets were not significant. MPA abrogated *in vitro *proliferation of purified B cells completely but had only moderate impact on B cell survival.

**Conclusions:**

The thorough inhibition of B cell activation and plasma cell formation by MMF might explain the favorable outcomes of previous clinical trials in patients with SLE, since enhanced B cell proliferation is a hallmark of this disease.

## Introduction

Systemic lupus erythematosus (SLE) is characterized by enhanced B cell proliferation and formation of antibody secreting cells (ASCs), therefore innovative therapeutic strategies target those cells. However selective targeting is a goal not always met. Memory B cells and ASCs have been shown to be less sensitive to cyclophosphamide [1] or belimumab [2] than antigen-naïve B cell subsets. In addition, it has been demonstrated that rituximab [3] and cyclophosphamide [4] spare long-lived plasma cells. Very little is known about the impact of other drugs used to treat or to prevent lupus flares on certain B cell subsets. Mycophenolate mofetil (MMF) is a drug used as a standard treatment especially in patients with lupus nephritis. It is as effective as cyclophosphamide in inducing remission [5], and in the long run even more effective than azathioprine (AZA) in maintaining remission of lupus nephritis [6]. Furthermore, side effects, such as cytopenia or infertility are less frequently observed in patients treated with MMF compared to cyclophosphamide [7].

Although the efficacy and safety of MMF have been thoroughly investigated in patients with severe SLE [5,8-10], the impact of MMF on B cell aberrations identified in lupus patients at a time before B-cell depletion came into fashion [11,12] has not yet been specifically addressed.

MMF is a pro-drug of mycophenolic acid (MPA). MPA reversibly inhibits inosine monophosphate dehydrogenase (IMPDH). MPA preferentially inhibits type-II-IMPDH that is upregulated in activated lymphocytes [13-15]. MMF was approved to prevent rejection in kidney allograft recipients in 1995 [16], but it is increasingly used in patients with autoimmune diseases because of a relatively high benefit-risk ratio. Since activated lymphocytes depend on type-II-IMPDH, MPA can theoretically selectively target these cells mediated by a depletion of the guanosine pool and deoxyguanosine triphosphate levels, resulting in cell cycle arrest. This might reduce the probability of side effects as compared to cytotoxic or other anti-proliferative drugs such as cyclophosphamide or AZA [17].

However, recent studies have revealed an impact of MPA on dendritic cells (DCs). Decreased surface expression of co-stimulatory molecules and a modification of DC/T cell interaction have been reported [18-20]. Those might contribute to its positive effects in preventing allograft rejection or nephritis flares. Furthermore, MPA might show alternate modes of action besides mere guanine nucleotide deprivation. In this context, signal transducer and activator of transcription 3 (STAT3) phosphorylation that has been shown to be required for memory B cell and ASC formation [21,22] seems to be impaired by MPA in myeloma cells [23]. Also IL-2-induced STAT5 phosphorylation is altered in MPA-treated CD3/CD28-activated T cells [24].

Although, there are almost no data available on the effect of MMF on lupus-specific alterations of DC or lymphocyte subsets, the effect of MMF on autoimmune mouse strains, such as MRLlpr/lpr and NZB/W mice, has been studied thoroughly. An improvement of lupus-related findings is observed when mice are exposed to MMF [25-29]. MRLlpr mice are characterized by enhanced B cell proliferation and extrafollicular differentiation of ASCs [30]. IL-21 has been shown to promote GC-derived [31] and extrafollicular [32] B cell proliferation and might therefore contribute to the generation of self-reactive ASCs in autoimmune mouse strains such as MRL/lpr mice. Therefore, we chose IL-21 to perform functional assays.

Combining observational data obtained by monitoring patients with SLE, and results of selected functional assays, this work develops an idea of how profoundly MPA acts in patients with SLE. It suggests modes of action that are advantageous especially in lupus patients.

## Materials and methods

### Patients

Data were obtained from outpatients attending a lupus clinic that was started in January 2010 as a joint initiative of nephrologists and rheumatologists at Münster University Hospital. All patients underwent standard laboratory tests allowing an assessment of disease activity as well as the safety of their current therapeutic regimen. These tests include all routine diagnostic procedures and established biomarkers, allowing an assessment of disease activity, and the patient stratification needed to decide on the most suitable therapeutic approach. Although no trial was planned, a characteristic pattern of B cell subsets became apparent in patients on MMF. A retrospective analysis of clinical, serological and cellular parameters was performed to compare patients taking MMF with patients taking AZA, and patients not taking immunosuppressive treatment. Ethical approval and informed consent for monitoring as well as retrospective data analysis were waived by the local ethics committee. All patients attending the clinic within its first year were included in this analysis, provided they had been taking their current medication for at least six months. Patients treated with rituximab at any time were excluded, since it causes a sustained alteration of B cell subsets.

Patients were between 18 and 72 years old (mean ± SD, 37.7 ± 12.8), mainly female (85.3%), and fulfilled the American College of Rheumatology (ACR) criteria for classification of SLE [33,34]. Regarding immunosuppressive therapy, three groups of patients were distinguished, namely patients taking AZA (*n *= 30) or MMF (*n *= 39), or not taking any immunosuppressive drugs (*n *= 38). All three groups comprised patients suffering flares as well as patients in remission. Disease activity, individual autoantibody profiles, and organ involvement were recorded. Given the current situation of off-label prescription of MMF in patients with SLE, patients taking MMF were slightly younger than the remaining patients (MMF, mean 34.7 ± 11.2 vs. AZA, 38.2 ± 13.3 years, or vs. no immunosuppressive therapy, 40.4 ± 13.5 years). Half the patients taking MMF had been receiving it as induction or maintenance therapy for lupus nephritis since adolescence, initially prescribed by their pediatricians. The remaining patients on MMF had AZA-refractory disease or contraindications to receiving cyclophosphamide or AZA. Table 1 gives an overview of the patient characteristics.

**Table 1:** Summary of additional medication, clinical and serologic characteristics of the analyzed lupus patients

	AZA		MMF		No IS	
	*n *= 30	%	*n *= 39	%	*n *= 38	%
**Medication **^a^						
Prednisone	26	87	38	97	14	37
mg/day, median (range)	5.0 (0-50) ^§§§^		5.0 (0-30)^###^		0 (0-20)^§§§ ###^	
Hydroxychloroquine	17	57	26	67	29	76
						
**Manifestations **^a^						
Nephritis	20	67	35	90	13	34
Proliferative class III, IV of all biopsy results available	12	75	22	76	3	38
CRF grade III+	5	25	6	17	1	8
Nephritis in remission	9	45	20	57	7	58
Active nephritis	11	55	15	43	6	46
New nephritis	1	5	0	0	6	46
Arthritis	3	10	2	5	9	24
Serositis	1	3	0	0	4	11
Myositis	2	7	1	3	0	0
CNS	1	3	0	0	1	3
Vasculitis	1	3	1	3	1	3
						
**Flares**^a^	14	47	9	23	12	32
Lupus nephritis flare	10	71	4	44	6	50
CNS flare	0	0	0	0	1	3
Arthritis flare	0	0	2	22	5	42
Serositis flare	2	14	0	0	3	8
Myositis flare	1	7	1	11	0	0
Vasculitis flare	0	0	1	11	0	0
Ref. thrombocytopenia	0	0	1	11	0	0
ILD flare	1	7	0	0	0	0
						
**Autoantibodies **^a^						
Anti-dsDNA	27	90	36	92	28	74
C _median_, U/ml (range)	39 (0-1,250)		29 (0-1,065)		32 (0-1,496)	
Anti-Ro	17	57	16	41	24	65
Anti-La	8	27	6	15	10	27
Anti-U1RNP	11	37	14	36	20	54
Anti-SM	6	20	6	15	8	22
APLA/LA	10	33	14	38^b^	9	28^b^
						
**Consumption of complement factors **^a^	15	50	16	41	15	39
						
**SLEDAI**						
Median (range)	6 (0-18)		4 (0-14)		4 (0-24)	

Results are presented as number and percent of patients unless otherwise stated. ^a^Resulting in an intensification of the immunosuppressive therapy (> 50mg prednisone-equivalent added, or switch of the therapy regimen to a more potent regimen, such as cyclophosphamide). AZA, azathioprine; MMF, mycophenolate mophetil; IS, immunosuppression; CRF, chronic renal failure; CNS, central nervous system; ILD, interstitial lung disease; APLA, anti-phospholipid antibodies; LA, lupus anticoagulant; SLEDAI, SLE disease activity index. ^b^Data incomplete (antibody status known in 37 and 32 patients respectively). Statistically significant differences detected between the dose of prednisone-equivalent, Dunn's Multiple Comparison test: ^#^*P *< 0.05, ^##^*P *< 0.01, ^###^*P *< 0.001 statistically significant differences between patients taking MMF compared to patients without immunosuppressive therapy (no IS). ^§^*P *< 0.05, ^§§^*P *< 0.01, ^§§§^*P *< 0.001 for statistically significant differences between patients taking AZA compared to patients without immunosuppressive therapy.

### Flow cytometric analysis of peripheral blood lymphocyte subsets

Flow cytometric analysis of peripheral blood mononuclear cells (PBMC) is a procedure performed routinely in addition to differential blood count in patients undergoing immunosuppressive therapy. From 3 ml of heparinized blood PBMCs were isolated by density gradient centrifugation using Ficoll-Paque™ Plus from GE Healthcare (Munich, Germany), were washed in PBS/0.5% BSA (Sigma-Adlrich, St. Gallen, Switzerland) and stained immediately with fluorochrome-labeled monoclonal antibodies to a panel of different surface antigens to discriminate B and T cell subsets (see Additional file 1). All samples were processed and analyzed within 6 hours after collection to ensure viability of all cell subsets. To exclude dead cells a final concentration of 220 nM 4´,6-diamidino-2-phenylindole (DAPI) (Invitrogen, Carlsbad, CA, USA) was used. A FACS Canto-II and FACS Diva Software (Becton Dickinson, (BD), San Jose, CA, USA) were used for 12-parameter (8-color) flow cytometric analysis. One million events were recorded for B cell and 500,000 events for T cell analysis. Results were analyzed using FlowJo (Treestar, Ashland, OR, USA).

Lymphocyte counts were recorded and absolute numbers were calculated using the frequencies of T and B cells based on the lymphocyte gate and the lymphocyte count. Differential blood counts and all other lab values including autoantibody titers were determined in the central laboratory using accredited diagnostic procedures [35].

### Isolation of B cell subsets for functional analysis

Naïve and memory B cells from blood donors (leukocyte filters) were isolated in a multistep procedure. The use of leukocyte filters from healthy blood donors for *in vitro *assays was approved by the local ethics committee. First the filter content was incubated with RosetteSep B Cell Enrichment Cocktail (STEMCELL Technologies SARL, Grenoble, France) according to the manufacturer's' instructions, and density gradient centrifugation was performed. Isolated B cells were then labeled with anti-CD27-magnetic beads (Miltenyi Biotec, Bergisch Gladbach, Germany) and CD27^+ ^memory B cells were positively selected using a magnetic column and the Miltenyi Biotec protocol. Subsequently the remaining B cells were labeled with biotin-labeled anti-IgD antibody (IA6-2, BD, Bioscience, Franklin Lakes, NJ, USA). After washing cells twice with PBS/0.5%BSA, spreptavidin-labeled magnetic beads (Miltenyi Biotec) were used for positive selection of CD27^-^IgD^+ ^naïve B cells on a second column.

Flow cytometric analysis after magnetic activated cell sorting (MACS) confirmed that CD3^+ ^T cells were depleted completely from the CD27^+ ^subset.

### Proliferation assay

Naïve and memory B cells were obtained as described above and subsequently stained with carboxyfluorescein-succinimidyl-ester (CFSE, Invitrogen) according to the manufacturers' instructions. CFSE-labeled cells were then incubated at a concentration of 10^5^/ml in RPMI 1640 (GIBCO, Invitrogen) supplemented with 10% v/v FCS, penicillin (100 U/ml) and streptomycin (100 mg/ml) (all Invitrogen) on a 96-well round-bottom plate (Greiner Bio-One, Kremsmuenster, Austria) with CpG oligodeoxynucleotide 2006, sequence: 5'-TsCsg sTsCsg sTsTsT sTsgsT sCsgsT sTsTsT sgsTsC sgsTsT-3' 2.5 µg/ml (TIB MolBiol, Berlin, Germany) or 0.5 µg/ml of a monoclonal anti-CD40 antibody (MAB89 (Beckmann Coulter, Eurocenter S.A., Nyon, Switzerland) and 50 ng/ml of IL-21 (GIBCO, Invitrogen) with or without 5 µM of MPA (Sigma-Aldrich). Four days later, cells were harvested, washed with PBS, and stained with monoclonal antibodies (see Additional file 1) in a 96-well v-bottom plate (Brand, Wertheim, Germany). Proliferation assays were performed in doublets using peripheral B cell subsets from four healthy blood donors. Flow cytometric analysis and data processing were performed as described.

### Determination of STAT3 phosphorylation by phospho-flow cytometry

PBMC were isolated by density gradient centrifugation and incubated with 5 µM MPA, or without MPA, for 24 hours in RPMI1640 supplemented with 10%v/v FCS, penicillin (100 U/ml) and streptomycin (100 mg/ml). Subsequently cells were stimulated with IL-21 (50 ng/ml) for 15 minutes at 37°C and fixed in 1.5% formaldehyde (ROCKLAND Gilbertsville, PA, USA) for 10 minutes at room temperature. Afterwards PBMC were washed in PBS/0.5% BSA and stained with fluorescence-labeled monoclonal antibodies to a panel of different surface antigens to allow the discrimination of B and T cell subsets (see Additional file 1). After another washing step, cells were incubated with methanol (ice-cooled, 100%, Riedel-de-Haen AG, Seelze, Germany) for 10 minutes at 4°C followed by two more wash steps. Next intracellular labeling of pSTAT3 was performed incubating cells with a monoclonal antibody (see Additional file 1) for 30 minutes at 4°C. All samples were acquired immediately after two more wash steps using a FACS Canto-II equipped with FACS Diva Software (BD) and data were subsequently analyzed using FlowJo software (Treestar). One million events were recorded.

### Statistical analysis

Frequencies of lymphocyte subsets analyzed *ex vivo *or *in vitro *were calculated using FlowJo software (TreeStar). Differences in frequencies or numbers of certain cell subsets were determined using the Kruskal-Wallis test and Dunn's multiple comparison test, since the majority of data were not normally distributed. Except for age (presented as mean ± SD), median values with the range are shown. The Chi-square test was used to determine if organ involvement, co-medications or disease flares were significantly over-, or under-represented in any of the patient cohorts. The Wilcoxon matched-pairs signed rank test was performed to compare *in vitro *cell survival with and without MPA. *P*-values < 0.05 were considered statistically significant. Data were analyzed using GraphPad Prism5 (GraphPad, San Diego, CA, USA).

## Results

### Clinical aspects

Although AZA- and MMF-treated patients had not been randomized for important parameters such as disease activity, prednisone-equivalent dose, disease manifestations or autoantibody profile, there were no significant differences in the occurrence of any parameter analyzed as shown in Table 1.

Disease flares requiring intensification of the current treatment regimen by increasing the prednisone-equivalent dose to > 50 mg/day and/or the dose, or type of immunosuppressant, were observed more often in patients taking AZA (47%) compared to MMF-treated patients (23%). Organ involvement observed during flares is shown in Table 1.

### Effect of AZA and MMF on peripheral blood lymphocytes

**Figure 1:**
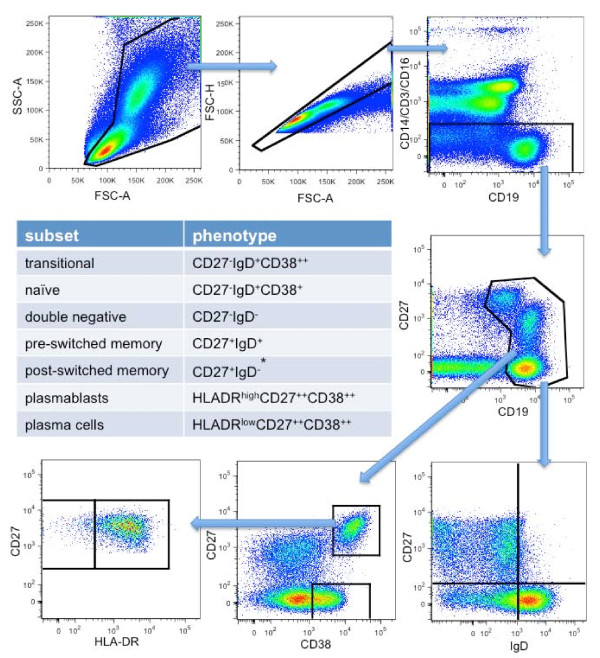
**Gating strategy**. After excluding doublets, CD3, CD14 and CD16-positive cells, CD19^+ ^cells were gated. *Antibody secreting cells (CD27^high^CD38^high ^plasmablasts and plasma cells) were subtracted from CD27^+^IgD^- ^B cells to calculate post-switched memory B cell frequencies.

**Figure 2:**
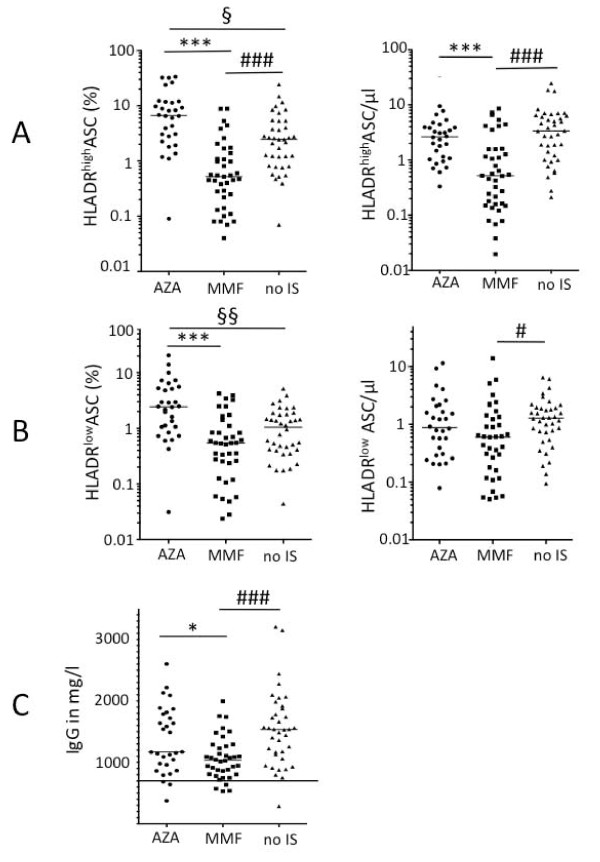
**Frequencies and absolute numbers of ASC subsets und IgG serum levels**. (**A**) Frequencies and numbers of HLADR^high ^antibody-secreting cells (ASC) were significantly lower in systemic lupus erythematosus (SLE) patients taking mycophenolate mofetil (MMF) compared to patients on azathioprine (AZA) and patients without immunosuppressive medication (no IS). (**B**) The absolute number of HLADR^low ^ASC was significantly lower in patients on MMF compared to patients without immunosuppressive therapy, whereas the frequency was not. (**C**) IgG serum levels (in mg/dl) were also significantly lower in patients taking MMF compared to patients on AZA or without IS. Lower limit of normal range is marked. Median values are shown: statistically significant differences were detected comparing patients on AZA and MMF (*P < 0.05, **P < 0.01, ***P < 0.001) or patients on AZA (^§^*P *< 0.5, ^§§^*P *< 0.01, ^§§§^*P *< 0.001) or patients on MMF (^#^*P *< 0.05, ^##^*P *< 0.01, ^###^*P *< 0.001) to patients without immunosuppressive therapy (Dunn's multiple comparison test).

**Figure 3:**
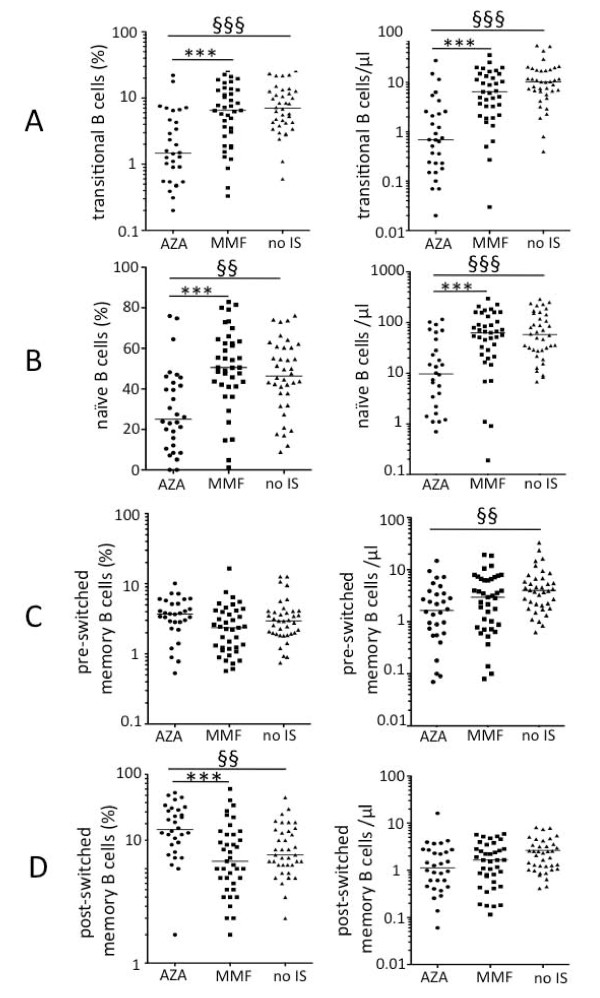
**Frequencies and absolute numbers of B cell subsets**. Frequencies and numbers of CD27^-^IgD^+^CD38^++ ^transitional (**A**) and CD27^-^IgD^+^CD38^+ ^naïve B cells (**B**) were significantly lower in patients taking azathioprine (AZA) compared to mycophenolate mofetil (MMF)-treated patients or patients not taking immunosuppressive therapy (no IS). Absolute numbers of CD27^+^IgD^+ ^pre-switched memory B cells (**C**) were significantly lower in patients taking AZA compared to patients without IS. However, absolute numbers of CD27^+^IgD^- ^post-switched memory B cells did not differ significantly (**D**). Patients taking AZA had the highest, and patients taking MMF the lowest frequency of CD27^+^IgD^- ^post-switched memory B cells (**D**). Median values are shown: statistically significant differences were detected comparing patients on AZA and MMF (*P < 0.05, **P < 0.01, ***P < 0.001) or patients on AZA (^§^*P *< 0.5, ^§§^*P *< 0.01, ^§§§^*P *< 0.001) or patients on MMF (^#^*P *< 0.05, ^##^*P *< 0.01, ^###^*P *< 0.001) to patients without immunosuppressive therapy (Dunn's multiple comparison test).

Differential blood counts and flow cytometric data were analyzed to estimate and compare the effect of AZA and MMF on cellular parameters. A summary of cell counts is shown in Table 2. Although patients without immunosuppressive treatment had higher T cell numbers than patients treated with MMF or AZA, no significant differences were observed. T cell subset analysis did not reveal significant differences between AZA- and MMF-treated patients and patients without immunosuppressive therapy. However, when B cell subsets were compared, a couple of significant differences were identified. Performance and results of the B cell subset analysis are shown in Figures 1, 2, 3.

**Table 2:** Summary of all cell counts recorded

	**AZA**	**MMF**	**No IS**
**Hemoglobin, mmol/l**	7.9 (5.5-10.4)	8.2 (5.4-10.7)	8.2 (4.5-10.7)
**Platelets/µl**	238 (48-459)	252 (136-404)	223 (96-379)
**Leukocytes**	4855 (2,060-10,160)	5970 (2,640-15,550)	5225 (2,080-9,050)
**Lymphocytes/µl**	987 (200-3,790)	1080 (360-3,010)	1330 (300-2,910)
**B cells %**	4.5 (0.6-31.3)* ^§§§^	9.4 (0.6-40.1)*	11.8 (3.6-28.9)^§§§^
**B cells/µl**	44(3-662)** ^§§§^	113(4-462)**	119 (40-460)^§§§^
**T % of L**	76 (28-93)*	63 (24-85)*	64 (42,2-84,5)
**T/µl**	584 (180-3,286)	640 (89-2,468)	807 (162-2,104)
CD4^+ ^%	73 (39-82)	71 (41-92)	69 (33-92)
CD4^+^/µl	430 (85-1,709)	466 (61-2,021)	518 (106-1,514)
CD8^+ ^%	19 (7-46)	22 (3-48)	22 (2-60)
CD8^+^/µl	127 (25-1,410)	144 (12-696)	207 (19-604)
CD4^-^/CD8^- ^%	6 (2-18)	6 (2-11)	5 (2-20)
CD4^-^/CD8^- ^/µl	41 (8-136)	41 (2-139)	46 (4-137)

Results are presented as median (range). Statistically significant differences detected between cell counts of patients taking azathioprine (AZA) and mycophenolate mofetil (MMF) (*P < 0.05, **P < 0.01, ***P < 0.001) and AZA compared to patients not taking immunosuppressive therapy ^§^*P *< 0.05, ^§§^*P *< 0.01, ^§§§^*P *< 0.001 (Dunn's multiple comparison test).

Although patients on AZA had significantly lower B cell counts compared to untreated patients or patients taking MMF (*P *= 0.0001) (Table 2), CD27^high^CD38^high ^ASCs were found at a significantly higher frequency and similar number in the peripheral blood of these patients compared to patients without immunosuppressive therapy (data not shown). Importantly, patients taking MMF had significantly lower frequencies and numbers of ASCs in the peripheral blood compared to AZA-treated patients, or patients without immunosuppressive therapy (*P *< 0.0001 respectively) (data not shown).

In order to analyze if the impact of MMF on the ASC subset is restricted to recently produced HLA-DR^high ^plasmablasts that usually represent the vast majority of the CD27^high^CD38^high ^ASC subset in patients with flaring lupus, peripheral HLA-DR^low ^ASC and HLA-DR^high ^ASC were determined and compared (Figure 2). Although the absolute number of HLA-DR^low ^ASC was significantly lower in patients on MMF (*P *= 0.017, Figure 2B), this deviation was moderate compared to the marked difference of HLA-DR^high ^ASC counts or frequencies observed between patient groups (*P *< 0.0001, Figure 2A). Therefore, MMF treatment was primarily associated with decreased frequencies and numbers of HLA-DR^high ^ASC in the peripheral blood of lupus patients.

Frequencies and numbers of CD27^-^IgD^+^CD38^++ ^transitional B cells were significantly lower in patients taking AZA compared to MMF-treated patients, or patients without immunosuppressive therapy (*P *< 0.0001, Figure 3A). In addition, frequencies and numbers of CD27^-^IgD^+^CD38^+ ^mature naïve B cells were significantly lower in patients taking AZA compared to MMF-treated patients, or patients without immunosuppressive therapy (*P *= 0.0001 and *P *< 0.0001, respectively, Figure 3B).

In contrast, memory B cell analyses revealed no difference, or less pronounced differences, between patient groups (Figures 3C and 3D). Median frequencies of CD27^-^IgD^- ^B cells (heterogeneous subset also containing memory B cells [36]) were comparably high in patients on MMF (16.2%, range 5.7 to 41.7%), AZA (17.7%, 7.7 to 36.1%) and patients not on immunosuppressive drugs (17.0%, 4.0 to 33.0%) (data not shown). Also median frequencies of CD27^+^IgD^+ ^pre-switched memory B cells did not differ (Figure 3C). The median frequencies of CD27^+^IgD^- ^post-switched memory B cells were comparably high in patients on MMF and patients not on immunosuppressive drugs, whereas AZA-treated patients had significantly higher frequencies of post-switched memory B cells (*P *= 0.0003). This comparative increase was caused by a lack of antigen-naïve B cells observed in AZA-treated patients. However, absolute numbers of post-switched memory B cells were comparably high in all patients (Figure 3D).

### Effect of MMF on IgG levels

Consistent with the results of B cell subset analysis and dsDNA antibody levels, patients taking MMF had significantly lower IgG levels compared to patients taking AZA, or patients not on immunosuppressive drugs (*P *= 0.0005, Figure 2C). Notably, no significant difference was found between the serum IgG levels of patients on AZA and patients not taking immunosuppressive drugs (Figure 2C). However the percentage of patients below the normal range was comparable in AZA- and MMF-treated patients (both 10.3%).

### *In vitro *B cell proliferation and differentiation of ASCs are abolished completely by MPA

B cell proliferation assays were performed as described to investigate if the impact of MMF on B cell activation and generation of ASCs that we postulated in patients with lupus, was indirect or direct. The MPA concentration that was used in all functional assays had been chosen considering previous work, and the approximate plasma concentration reached by daily intake of 2-3 g MMF [37].

**Figure 4:**
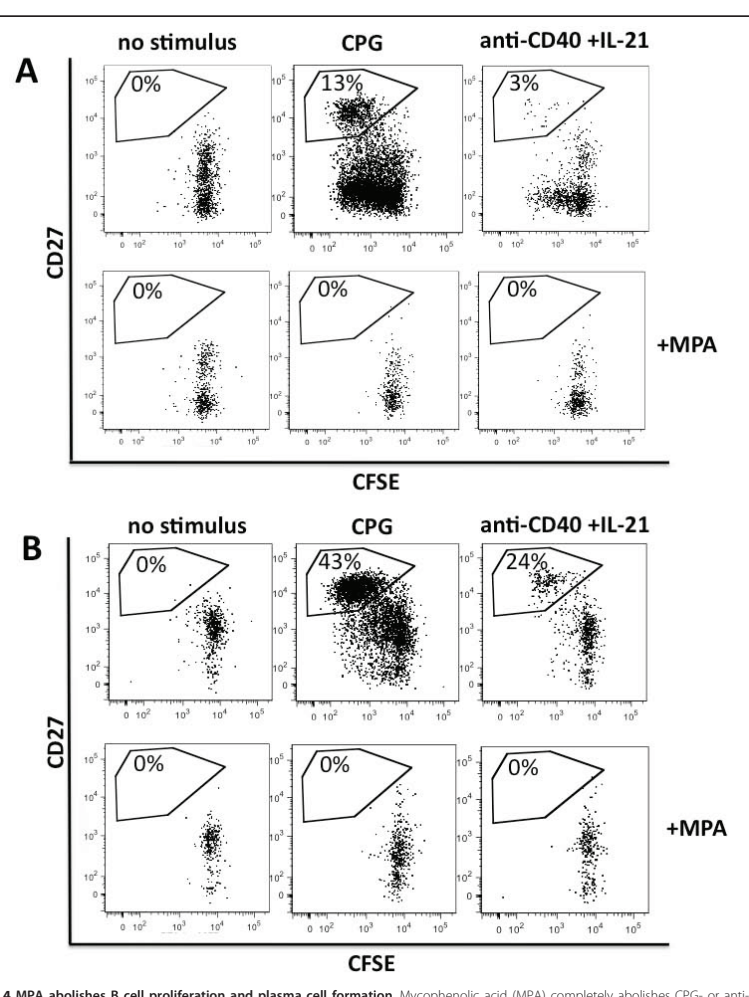
**MPA abolishes B cell proliferation and plasma cell formation**. Mycophenolic acid (MPA) completely abolishes CPG- or anti-CD40 +IL21-induced proliferation of CD27^-^antigen-naïve B cells (**A**) or CD27^+ ^memory B cells (**B**). Results of a representative B cell proliferation assay of a healthy donor are shown. CD27^-^IgD^+ ^B cells (**A**) or CD27^+ ^B cells (**B**) were isolated, labeled with carboxy-fluorescein-succinimidyl ester (CFSE) and stimulated for 3 to 4 days as indicated. Gated on live cells, plasmablast gates are shown. Dividing-B cells lose CFSE with any cell division.

Proliferation assays of purified CD27^-^IgD^+ ^antigen-naïve and CD27^+ ^memory B cell subsets from the peripheral blood of four healthy blood donors were performed. All individuals showed a comparable pattern of proliferation and a representative example is shown (Figure 4). In all assays MPA was able to abolish B cell proliferation and differentiation of ASCs completely, no matter if it was caused by TLR-mediated stimulation using CpG, or by CD40-antibodies in combination with IL-21, suggesting that the impact of MMF on B-cell activation and proliferation is not only related to impaired T cell help. In contrast to calcineurin inhibitors [38], MMF inhibits B cell activation and differentiation of ASCs directly.

While proliferation and formation of ASCs were abolished completely, the survival of non-stimulated B cells was only partially impaired by 5µM MPA after 4 days of culture (Figure 4). Comparing cell survival in cultures incubated with and without MPA it was reduced to a median value of 52.4% for antigen-naïve (range 34.9 to 67.8, *P *= 0.125) and 76.4% for CD27^+ ^memory B cells (range 51.1 to 82.3, *P *= 0.125 (data not shown).

### MPA does not affect STAT3 phosphorylation in antigen-naïve or CD27^+ ^memory B cells

**Figure 5:**
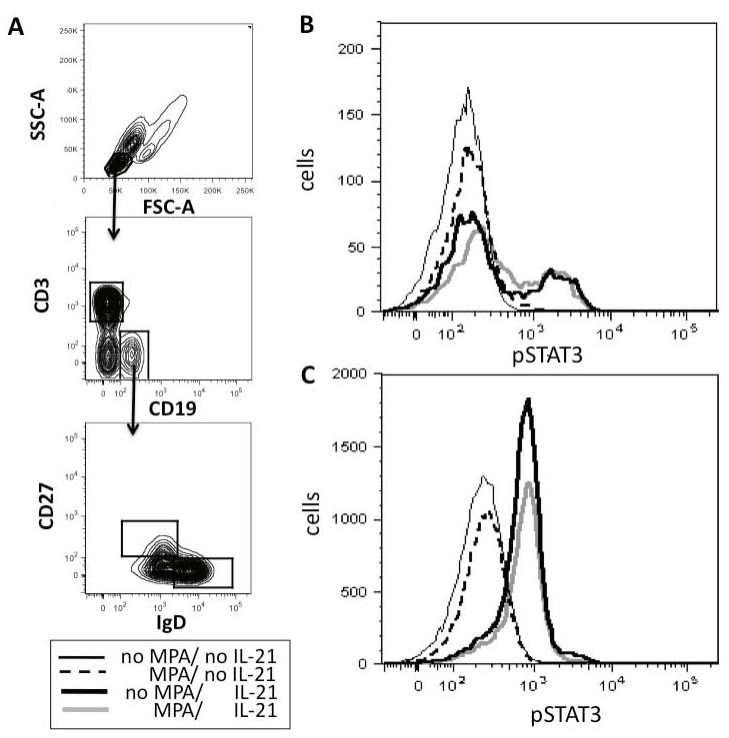
**Mycophenolic acid (MPA) does not impact on IL-21-induced signal transducer and activator of transcription 3 (STAT3) phosphorylation in antigen-naïve and CD27^+ ^memory B cells**. After pre-incubating peripheral blood mononuclear cells (PBMC) for 24 hours with or without MPA, cells were exposed to IL-21 for 15 minutes. STAT3-phosphorylation was determined by phospho-flow cytometry. Gating procedure of CD19^+ ^B cells and CD27^+^IgD^- ^memory B cells and CD27^-^IgD^+ ^antigen-naïve B cells (**A**) and pSTAT3 of CD27+ memory (**B**) and CD27^-^IgD^+ ^antigen-naïve B cells (**C**) are shown.

To rule out additional effects of MMF on mechanisms that are important for B cell proliferation and ASC formation we also analyzed STAT3 phosphorylation. STAT3 signal transduction is required to form memory B cells and ASCs [21,22]. It is essential for humoral immune responses. Phosphorylation of STAT3 has been shown to be impaired in myeloma cells after incubation with MPA [23]. Therefore, we decided to investigate the impact of MPA on IL-21-induced STAT3 phosphorylation in antigen-naïve and CD27^+ ^memory B cells from the peripheral blood of healthy individuals. As shown in Figure 5, a 24-hour pre-incubation with 5 µM MPA did not impact STAT3 phosphorylation detected after exposure of CD27^-^IgD^+ ^antigen-naïve or CD27^+ ^memory B cells to IL-21.

## Discussion

Treatment of lupus patients with MMF causes an improvement of symptoms as well as normalization of paraclinical aberrations, such as increased peripheral plasmablast counts or hypergammaglobulinemia. Its preferential effect on B cell activation, proliferation and plasma cell generation is most welcome in patients with SLE and seems to be associated with high therapeutic efficacy combined with favorable safety. While controlled clinical trials have recently demonstrated the latter, this study focused on paraclinical aspects. Combining observational data and results obtained by performing selected functional assays it suggests modes of action that are especially advantageous in lupus, a disease characterized by enhanced B cell activation and proliferation, as well as plasma cell expansion and autoantibody secretion.

While the beneficial effects of MMF have already been described years ago in autoimmune mouse models [25-28] and its impact on aberrations of lymphocyte subsets has been analyzed in mice [29], data on the impact of MMF on lymphocyte subset alterations in patients with SLE are rare. Although the current study has limitations because of its observational character, and restrictions with regard to the material investigated (blood), the results still provide explanations for the beneficial effects observed *in vivo*. In contrast to AZA or cyclophosphamide [1] MMF shows a more selective mechanism of action. It counteracts the enhanced B cell activation that is often observed in patients with SLE. In contrast to AZA, rituximab and cyclophosphamide MMF seems to spare antigen-naïve B cell subsets. This could be advantageous in infections, when pausing MMF might allow humoral immune responses to occur. We did not observe an influence of MPA on resting memory B cells. Compared to the effects observed on B cells, the influence of MMF on T cell subsets seems to be minor in patients with SLE. However, comparing peripheral blood T cell counts and subsets has limitations. Endothelial adhesion of T cells is impaired by MPA [39]. Therefore, T cell numbers and subsets in the peripheral blood might not necessarily reflect the situation found in secondary lymphoid organs or inflamed tissue; there, T cell-dependent antibody responses take place and they might be influenced by MPA. Although, the results of the current study point to a predominant and direct inhibition of B cell proliferation and formation of plasmablasts by MPA, they do not preclude additional effects of this drug on T cells or DCs.

Until recently, the impact of MMF on human B cell subsets has been neglected, because T lymphocytes enjoyed unshared attention as key players of allograft rejection. A study published a few years ago addressed the *in vitro *effect of MPA on purified total human B cells and described an inhibition of CD40-induced proliferation [40]. Another study investigated the effect of starting MMF, on peripheral B and T cell activation markers in 10 patients with SLE [41] and found a decrease of CD38^++^CD19^+ ^B cells in most patients.

In agreement with our results it has been shown very recently that B cell proliferation and differentiation of plasma cells is inhibited by MPA even at very low concentrations (0.3µM), not affecting cell survival [42]. In contrast to B cells, terminally differentiated plasma cells were unresponsive to MPA even at very high concentrations, because of low type-II-IMPDH expression [42]. In line with this lack of impact on long-lived plasma cells, the HLA-DR^low ^ASC subset was only modestly diminished in patients on MMF compared to HLA-DR^high ^plasmablasts that were markedly lower in patients taking MMF compared to patients on AZA, or patients without immunosuppressive therapy. However, long-term use of MMF might compromise or skew plasma cell memory by lacking influx of newly generated plasma cells into the bone marrow. Although data on vaccination are still limited, humoral immune responses to influenza vaccination seem to be impaired more markedly by MMF than by AZA in kidney transplant recipients and patients with SLE [43,44]. A defect of humoral immune responses that was associated with infections was also identified in patients on MMF for prevention of renal allograft rejection [45].

Even if hypergammaglobulinemia is frequently observed in lupus patients and low IgG levels that are associated with infectious complications are rare, the data suggest that close monitoring of IgG levels is reasonable in patients on MMF, especially when combinations of immunosuppressive drugs are required or significant proteinuria is present. In addition, flow cytometric monitoring of B cell subsets might help to access MMF efficacy when starting or weaning off treatment, and to identify non-adherent or unresponsive patients with type-II-IMPDH polymorphisms [46].

Summarizing previous and current *in vitro *results, MPA seems to act directly on antigen-naïve and memory B cells, probably mediated by guanosine nucleotide deprivation. An additional impact on STAT3 phosphorylation as observed in a myeloma cell line [23] has not been detected in human B cells. MPA completely abolished TLR-mediated polyclonal B cell proliferation, as well as CD40- and IL-21-induced proliferation. Moreover, alterations of DC or T cell subsets in patients on MMF could further contribute to the positive impact on B cell subsets observed in these patients, although those were not the subjects of this study.

Summarizing the observational data, MMF seems to be able to ameliorate characteristic disturbances of B cell subsets in patients with SLE, such as an increase in plasmablasts, or preferential depletion of antigen-naïve B cells, which is usually a result of AZA or cyclophosphamide treatment. These results are in line with the sustained clinical benefit and favorable safety profile observed in patients treated with MMF for induction or maintenance therapy of lupus nephritis or extrarenal manifestations.

## Conclusion

The thorough inhibition of B cell activation and plasma cell synthesis by MMF might explain the favorable outcomes of previous clinical trials in patients with SLE, since enhanced B cell proliferation is a hallmark of this disease. Considering the data obtained in this study and the results of previous randomized clinical trials we suggest that MMF should be used on a regular basis in patients with SLE, especially if signs of enhanced B cell activation are detected, and if maintenance therapy is required for several years, as in children or young adults with organ-threatening lupus nephritis.

## Abbreviations

ACR: American College of Rheumatology: ASC: antibody secreting cell; AZA: azathioprine; BSA: bovine serum albumin; CFSE: carboxy-fluorescein-succinimidyl ester; DAPI: 4´,6-diamidino-2-phenylindole; DC: dendritic cell; FCS: fetal calf serum; GC: germinal center; HLA: human leukocyte antigen; Ig: immunoglobuline; IL: interleukin; IMPDH: inosine monophosphate dehydrogenase; MMF: mycophenolate mofetil; MPA: mycophenolic acid; PBMC: peripheral mononuclear cells; PBS: phosphate buffered saline; SLE: systemic lupus erythematosus; STAT: signal transducers and activators of transcription.

## Competing interests

The authors declare that they have no competing interests.

## Authors' contributions

SE, EM, EJ, J-RN, and AMJ contributed to the data acquisition and analysis. SE, HJP, and AMJ contributed to the study design and manuscript preparation. All authors have read and approved the final manuscript for publication.

## Supplementary Material

Additional file 1 **Table S1**. The file contains detailed information about the monoclonal antibodies used for flow cytometric analysis. Click here for file
