# Supplementary material for: Mycophenolic acid counteracts B cell proliferation and plasmablast formation in patients with systemic lupus erythematosus
Source: Arthritis Res Ther. 2012 May 9;14(3):R110. doi: 10.1186/ar3835 (PMC4060361; doi:10.1186/ar3835)
Supplement: Additional file 1 — Table S1. The file contains detailed information about the monoclonal antibodies used for flow cytometric analysis. [file ar3835-S1.DOCX]

**Supplemental material**

Tabel S1, antibodies used for flow cytometrical analysis

| **target** | **clone** | **label** | **detected with** | **source** |
| --- | --- | --- | --- | --- |
| CD3 | UCHT1 | Pacific Blue |  | BD |
| CD3 | SK7 | PerCP |  | BD |
| CD4 | RPA-T4 | APC-H7 |  | BD |
| CD8 | RPA-T8 | V500 |  | BD |
| CD14 | M5E2 | Pacific Blue |  | BD |
| CD19 | HIB19 | Pacific Blue |  | eBioscience |
| CD19 | SJ25C1 | PE-Cy7 |  | BD |
| CD20 | L27 | V450 |  | BD |
| CD27 | L128 | APC |  | BD |
| CD56 | B159 | V450 |  | BD |
| CD38 | HB-7 | PE |  | BD |
| CD44 | G44-26 | PerCP-5.5 |  | BD |
| CD45RA | HI100 | PE-Cy7 |  | BD |
| CD45RO | UCHL1 | APC |  | BD |
| HLA-DR | TÜ36 | Pacific Orange |  | Invitrogen |
| CD62L | Dreg56 | FITC |  | BD |
| CD69 | FN50 | PE |  | BD |
| IgD | IA6-2 | biotin | SA-Alexa Fluor 680  SA-PE-Cy7 | BD/Invitrogen |
| pStat3 | 4/P-STAT3 | PE |  | BD |

PE: R-Phycoerythrin, PerCP: Peridinin chlorphyll protein, FITC: Fluorescein, APC: Allophycocyanin, SA: streptavidin
